# Prognostic Factors and Biomarker Performance in Patients with Colorectal Cancer Receiving Reduced-Dose 5-Fluorouracil Therapy: A Retrospective Cohort Analysis

**DOI:** 10.3390/jcm15010071

**Published:** 2025-12-22

**Authors:** Mei-Wen Chen, Jing-Jim Ou, Cheng-Shyong Chang

**Affiliations:** 1 Cancer Administration and Coordination Center, Chang Bing Show Chwan Memorial Hospital, Changhua 505, Taiwan; show15820@gmail.com; 2 Department of Information Management, National Open University, New Taipei 247, Taiwan; 3 Department of Colon and Rectal Surgery, Chang Bing Show Chwan Memorial Hospital, Changhua 505, Taiwan; 4 Department of Hematology and Oncology, Chang Bing Show Chwan Memorial Hospital, Changhua 505, Taiwan

**Keywords:** colorectal cancer, 5-fluorouracil, biomarkers, prognostic factors, survival analysis

## Abstract

**Background:** Patients with colorectal cancer (CRC) have varying responses to 5-fluorouracil (5-FU) treatment, particularly reduced-dose regimens. Inflammatory and tumor-associated serum biomarkers, such as carcinoembryonic antigen (CEA), carbohydrate antigen 19-9 (CA19-9), and cancer antigen 125, may refine prognostic assessment. However, their combined performance in patients with CRC receiving reduced-dose 5-FU remains understudied. This retrospective study evaluated the prognostic value of multiple biomarkers in these patients, aiming to identify optimal combinations for personalized therapeutic strategies and improved clinical outcomes. **Methods:** Data (2017–2023) on patients’ clinicopathological characteristics and pretreatment serum biomarker levels were collected from a medical center in central Taiwan. Dose classification followed institutional standards. Reduced-dose chemotherapy was confirmed from patients’ medical records. Intergroup comparisons, receiver operating characteristic curve analysis, logistic regression, Cox proportional hazards modeling, and survival analysis were performed. Furthermore, a multivariate prognostic nomogram was constructed. **Results:** The study cohort comprised 95 patients receiving reduced-dose 5-FU. Univariate analyses highlighted cigarette smoking, advanced stage, poor tumor differentiation, and elevated pretreatment CEA level as significant predictors of mortality. Multivariate analysis indicated tumor differentiation grade and pretreatment CEA level as significant independent predictors. Cancer antigen 125, CEA, and CA19-9 exhibited robust discriminatory performance. The multivariate nomogram exhibited acceptable discrimination. **Conclusions:** Tumor differentiation, disease stage, and pretreatment CEA level emerged as independent predictors of overall survival in patients with CRC receiving reduced-dose 5-FU. Serum biomarkers, particularly CEA and CA19-9, may be included in comprehensive prognostic models alongside clinicopathological characteristics. The validated prognostic nomogram may support personalized risk stratification and individualized dose-adjusted chemotherapy.

## 1. Introduction

Colorectal cancer (CRC) represents a major public health challenge worldwide due to its substantial morbidity and mortality rates. Epidemiological estimates suggest nearly 1.9 million new CRC cases globally in 2025, positioning it as the third most frequently diagnosed cancer. It is also the second leading cause of cancer-related deaths, contributing to more than 900,000 fatalities annually [1]. Marked sex disparities are apparent in incidence rates, with men exhibiting higher rates (23.4 per 100,000) than women (16.2 per 100,000); the global average is approximately 19.5 per 100,000 population. Mortality patterns mirror this difference, with standardized rates of 11.0 and 7.2 per 100,000 in men and women, respectively, for an overall mortality rate of 9.0 per 100,000 worldwide [2]. CRC mortality has increased in Taiwan. Annual deaths increased 4.3-fold from 1100 cases (standardized mortality rate: 9.9/100,000) in 1981 to 7007 cases (14.2/100,000) in 2023 [3]. Incidence rates have surged even more dramatically: from 4217 cases (19.38/100,000) in 1995 to 19,678 cases (84.18/100,000) in 2022, equating to a substantial 64.8% increase over the period [4].

5-Fluorouracil (5-FU) remains the cornerstone systemic chemotherapeutic agent for CRC, with clinical efficacy in both adjuvant and palliative settings. 5-FU inhibits DNA and RNA synthesis by targeting folate metabolism, which curtails cancer cell proliferation and induces programmed cell death. Its therapeutic efficacy, however, is critically limited by both innate and acquired chemoresistance; objective response rates are persistently low (10–15%) in advanced CRC [3]. For older adults or frail patients, clinical guidelines support dose-reduction strategies. Initiating treatment at 70–80% of the standard dose calculated based on body surface area (BSA) achieves comparable therapeutic plasma concentrations and maintains survival outcomes. The emergence of pharmacokinetic-guided individualized dosing paradigms further optimizes efficacy while minimizing toxicity risk. Conventional BSA-based dosing often fails to track plasma clearance rates, leading to wide interpatient variability in drug exposure, which can inadvertently result in subtherapeutic dosing or toxic overdosing. Because plasma 5-FU concentrations are monitored directly, pharmacokinetic-guided dosing can ensure that drug exposure is maintained within the therapeutic window for improved clinical outcomes [5,6,7,8]. However, routine implementation of TDM remains challenging in many clinical settings due to resource and logistical constraints. In practice, physicians often resort to empiric dose reductions based on clinical parameters—such as performance status and age—to mitigate toxicity risks in vulnerable populations. While TDM represents the pharmacological gold standard, the prognostic implications of such clinically guided reduced-dose strategies remain to be fully characterized.

Advancements in biomarker-driven approaches have transformed CRC management, enabling refined prognostic stratification and treatment response prediction. C-reactive protein (CRP), a well-established systemic inflammatory marker, is strongly associated with tumor CRC progression and recurrence risk, reflecting the inflammatory microenvironment that fosters cancer development and metastasis [9]. Similarly, the neutrophil-to-lymphocyte ratio (NLR), another systemic inflammatory marker, is correlated with poorer overall survival and disease-free survival in patients with CRC, indicating its role in reflecting the tumor-promoting inflammatory milieu that facilitates disease progression and metastasis [10]. However, the most extensively validated and clinically utilized tumor marker in CRC management is carcinoembryonic antigen (CEA). Elevated CEA levels are strongly correlated with advanced disease stage, poor histological differentiation, and unfavorable prognostic outcomes, rendering CEA indispensable for diagnostic evaluation, prognostic assessment, and longitudinal treatment monitoring [11]. Carbohydrate antigen 19-9 (CA19-9) serves as a valuable complementary biomarker to CEA, offering additional prognostic stratification related to both overall survival and recurrence-free survival [12]. Although primarily associated with ovarian malignancies, cancer antigen 125 (CA125) has demonstrated independent prognostic significance in CRC, occasionally exhibiting superior predictive capability compared with traditional CEA measurements [13]. Finally, alpha-fetoprotein (AFP)-producing CRC constitutes a rare but clinically significant aggressive subtype characterized by advanced-stage presentation and increased hepatic metastatic propensity. AFP positivity functions as a potent negative prognostic indicator, potentially mediated via dysregulated hepatocyte growth factor (HGF)/c-Met signaling pathways that enhance tumor aggressiveness and metastatic potential [14].

Integrating multiple biomarker panels allows for more nuanced patient stratification and better-informed treatment decisions. Nevertheless, the prognostic value of these panels in patients with CRC receiving reduced-dose 5-FU therapy is insufficiently explored. In the present study, we evaluated the prognostic impact of multiple biomarkers in patients with CRC treated with reduced-dose 5-FU, aiming to identify optimal combinations that facilitate individualized therapeutic strategies and improve clinical outcomes in this patient population.

## 2. Materials and Methods

### 2.1. Study Design and Data Extraction

For this retrospective cohort study, data of patients with CRC treated with reduced-dose 5-FU chemotherapy between January 2017 and December 2023 were extracted from the cancer registry database of Chang Bing Show Chwan Memorial Hospital, Taichung, Taiwan. These data included the patients’ demographic information, details of chemotherapy regimens and durations, laboratory test results, and records of treatment-related adverse events. Data collection focused on capturing the full spectrum of clinical, pathological, and biomarker information necessary for statistical analysis and prognostic modeling.

Biomarker levels were dichotomized as positive or negative based on the institution’s standard clinical reference ranges. Specifically, pretreatment CEA levels > 5 ng/mL, CA19-9 levels > 37 U/mL, CRP > 0.5 mg/dL, and CA125 levels > 35 U/mL were defined as positive. For other parameters, interpretations followed standard clinical guidelines. Patients were excluded from the study if they presented with multiple primary malignancies or were managed by physicians outside the study team.

To explicitly define the treatment context, the study population was categorized based on therapeutic intent and disease stage. Patients with Stage II (*n* = 10) and Stage III (*n* = 46) disease were treated with curative intent; their management primarily consisted of curative surgical resection followed by adjuvant systemic therapy (chemotherapy or concurrent chemoradiotherapy). Conversely, patients with Stage IV disease (*n* = 39) received treatment with palliative intent. This group comprised patients with de novo metastatic disease or those presenting with distant metastases at baseline. Their management was multimodal, including systemic chemotherapy, targeted therapy, and palliative surgical interventions (e.g., primary tumor resection, metastasectomy, or hyperthermic intraperitoneal chemotherapy) as clinically indicated.

The study protocol was approved by the Institutional Review Board of Show Chwan Memorial Hospital (SCMH IRB number 1140402). Because of the retrospective design and minimal risk due to the use of deidentified clinical data, the requirement for informed consent was waived.

### 2.2. Treatment Regimen and Dose Classification

At Chang Bing Show Chwan Memorial Hospital, the standard chemotherapy regimen for CRC comprises a combination of irinotecan, oxaliplatin, leucovorin, and 5-FU. Specifically, irinotecan (180 mg/m^2^) is diluted in 250 mL of normal saline, and oxaliplatin (85 mg/m^2^) is diluted in 250 mL of 5% dextrose, both administered through intravenous infusion over 2 h. Leucovorin (400 mg/m^2^) is then administered intravenously prior to 5-FU. Subsequently, 5-FU is delivered as a 400 mg/m^2^ intravenous bolus, followed by a continuous intravenous infusion of 2400 mg/m^2^ over 46–48 h.

All drug dosages are calculated according to the patient’s BSA. In this study, “reduced-dose 5-FU therapy” was defined as an upfront planned reduction in the starting dose of 5-FU (both bolus and infusion components), typically administered at 70–80% of the standard BSA-calculated dose. All included patients (*n* = 95) received reduced-dose 5-FU at treatment initiation; patients receiving full-dose 5-FU were not eligible for inclusion. While reductions could also extend to concomitant agents (oxaliplatin or irinotecan) depending on the patient’s condition, the classification criteria necessitated a reduction in 5-FU. These dose modifications were determined prior to treatment initiation based on clinical assessments, including Eastern Cooperative Oncology Group (ECOG) performance status, advanced age, renal or hepatic dysfunction, and comorbid conditions, rather than being reactive adjustments solely due to treatment-emergent toxicity. It is important to emphasize that therapeutic drug monitoring (TDM) was not performed in this cohort. Dosing decisions relied exclusively on standard BSA calculations adjusted by clinical judgment, without real-time pharmacokinetic guidance.

### 2.3. Statistical Analysis

Data management was performed in Microsoft Excel, and all analyses were conducted in R version 4.5.1 (R Foundation for Statistical Computing, Vienna, Austria) using RStudio version 2023.12.1 (Posit Software, PBC, Boston, MA, USA). Continuous variables are summarized as mean ± standard deviation (SD) and range and were compared using Student’s *t*-test or the Mann–Whitney U test, as appropriate, whereas categorical variables are summarized as counts and percentages and were compared using the chi-square test or Fisher’s exact test.

Receiver operating characteristic (ROC) analysis was used to evaluate biomarker diagnostic performance, and the area under the curve (AUC), sensitivity, specificity, positive predictive value, negative predictive value, and Youden’s index were used to determine optimal cutoffs.

Risk factors for mortality were examined with univariable and multivariable logistic regression, with results reported as odds ratios (ORs) with 95% confidence intervals (CIs). Overall survival was estimated using the Kaplan–Meier method and compared with the log-rank test. Independent prognostic factors for overall survival were identified using Cox proportional hazards regression, with hazard ratios (HRs) and 95% CIs reported. The proportional hazards assumption was assessed using scaled Schoenfeld residuals.

Model diagnostics included the assessment of multicollinearity through variance inflation factors and, where complete or quasi-complete separation was suspected, the application of Firth’s penalized likelihood logistic regression. All hypothesis tests were two-sided, with statistical significance defined as *p* < 0.05. Finally, a prognostic nomogram was constructed to predict 1-, 3-, and 5-year overall survival. The performance of the nomogram was assessed using the concordance index (C-index) and calibration plots. Internal validation was performed using bootstrap resampling to test the model’s reliability.

## 3. Results

**Figure 1:**
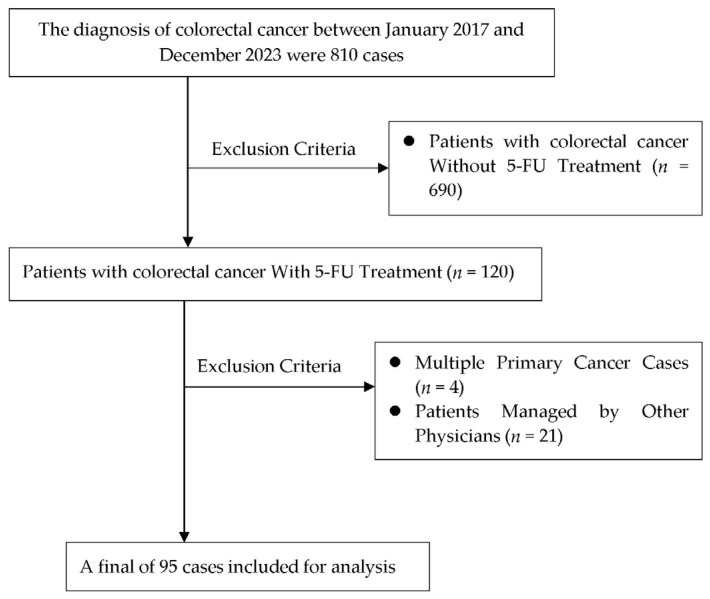
Flowchart for study design.

Of the 120 patients with CRC treated with 5-FU from January 2017 to December 2023 at Chang Bing Show Chwan Memorial Hospital, 25 were excluded based on the eligibility criteria. Eventually, 95 patients were included in the analysis (Figure 1).

As detailed in Table 1, the 95 patients included 55 men (57.9%) and 40 women (42.1%), with a mean age of 59.0 ± 10.7 years (range: 31–82 years). The primary tumor sites were the colon (67.4%), rectum (23.1%), and rectosigmoid junction (9.5%). Adenocarcinoma was the predominant histological subtype (100.0%). Clinical tumor differentiation was classified as well differentiated (25.3%), moderately differentiated (49.5%), and poorly differentiated (3.2%), whereas pathological evaluation indicated that most cases were moderately differentiated (80.0%). Perineural invasion was present in 64.2% of the patients, and lymphovascular invasion in 86.3%. Most patients were diagnosed at advanced stages, with 48.4% having stage III and 41.1% having stage IV CRC. In addition, 51.6% of the patients tested positive for pretreatment CEA. Genetic analysis in this cohort revealed KRAS mutations in 30.5% of the tested individuals, BRAF mutations in 7.4%, and microsatellite instability in 4.2%. Lifestyle risk factors included smoking (28.4%), alcohol consumption (17.9%), and betel nut chewing (8.4%). Clinical complications documented during the course of treatment included intestinal obstruction (14.7%) and perforation (1.1%).

Univariable logistic regression (Table 2) indicated that cigarette smoking was associated with a significantly higher mortality risk, with smokers showing a 2.9-fold increased odds of death compared with nonsmokers (OR: 0.343 for nonsmokers vs. smokers, 95% CI: 0.128–0.859, *p* = 0.022). Clinical moderate/poor differentiation vs. well differentiation was linked to 4.45-fold higher odds of death (OR: 4.452, 95% CI: 1.605–13.653, *p* = 0.004). Stage IV vs. II carried 5.18-fold higher odds (OR: 5.183, 95% CI: 1.279–23.086, *p* = 0.021). BRAF mutation vs. wild-type was also associated with increased mortality odds (OR: 6.810, 95% CI: 1.185–72.433, *p* = 0.030).

In the multivariable logistic regression analysis (Table 3), several associations reversed direction relative to the univariable analyses and yielded implausibly large effect sizes with exceptionally wide CIs. Betel nut chewing demonstrated a markedly inflated OR of 105.09 (95% CI: 2.44–7471.82, *p* = 0.018), cigarette smoking appeared spuriously protective (OR: 0.005, 95% CI: 0.000–0.108, *p* = 0.003), and positive pretreatment CEA—contrary to clinical expectations—was associated with lower mortality odds (OR: 0.109, 95% CI: 0.013–0.633, *p* = 0.023). These patterns are characteristic of sparse-data bias, quasi-complete separation, and/or multicollinearity, rendering the corresponding coefficients unsuitable for clinical interpretation; accordingly, we prioritized univariable and time-to-event Cox regression results for prognostic model development. The extreme estimates and wide intervals observed for betel nut chewing, smoking, lymphovascular invasion, and variables with non-trivial missingness (e.g., clinical and pathological grade, perineural invasion, MSI, and biomarkers) likely reflect small cell counts and separation. While these missing data were modeled as separate “not assessable” categories to maximize the sample size, we acknowledge that this approach contributes to unstable estimates. Therefore, these results should be interpreted with caution and, where appropriate, corroborated using Firth-penalized or exact logistic regression.

Note: Because of sparse-data bias and quasi-complete separation, several estimates in this multivariable model show extreme odds ratios with wide confidence intervals and should be interpreted with caution. Univariable analyses and Cox regression models (Table 2 and subsequent analyses) provide more reliable prognostic indicators. * Statistically significant (*p* < 0.05).

**Figure 2:**
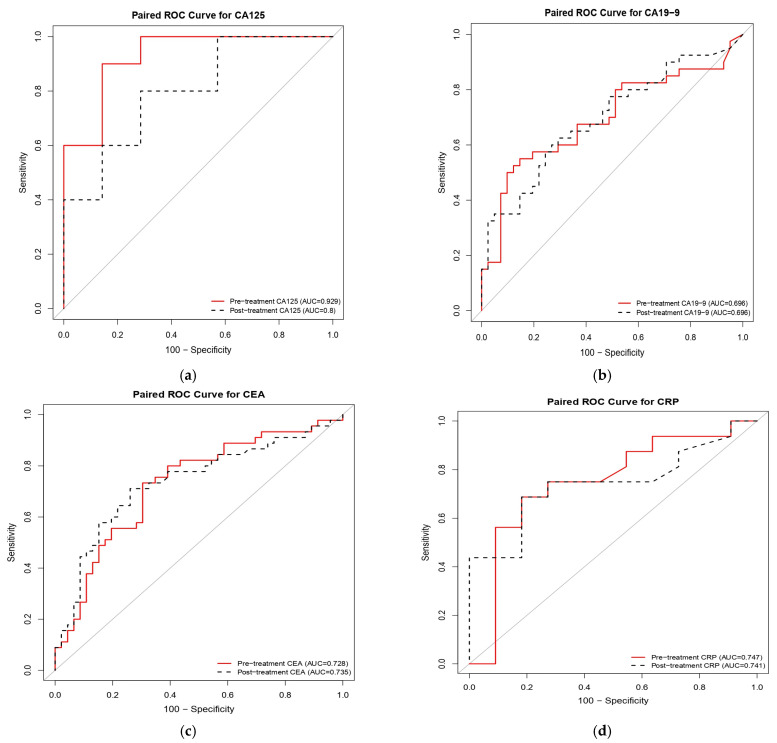
Receiver operating characteristic (ROC) curves comparing the prognostic performance of serum biomarkers at pretreatment and posttreatment time points in patients with colorectal cancer receiving reduced-dose 5-fluorouracil (5-FU). The panels illustrate the discriminatory ability for mortality risk of (**a**) cancer antigen 125 (CA125; *n* = 17), (**b**) carbohydrate antigen 19-9 (CA19-9; *n* = 81), (**c**) carcinoembryonic antigen (CEA; *n* = 91), and (**d**) C-reactive protein (CRP; *n* = 27). Red solid lines represent pretreatment values, while black dashed lines indicate posttreatment values. Pretreatment CA125 demonstrated the highest discriminative capacity (AUC = 0.929), whereas CEA and CA19-9 exhibited consistent performance across time points.

Table 4 summarizes the diagnostic performance of significant biomarkers in both pretreatment and posttreatment cohorts. The ROC curves comparing the discriminatory performance of these biomarkers are presented in Figure 2. In the pretreatment cohort, CA125 demonstrated the highest diagnostic accuracy in a limited subset (*n* = 17), with an AUC of 0.929 (*p* < 0.001) (Figure 2a), achieving 90.00% sensitivity and 85.71% specificity; however, the small sample size (*n* = 17) limits the generalizability of these results. By contrast, CEA (N = 91, AUC: 0.728) (Figure 2c) and carbohydrate antigen 19-9 (CA19-9) (*n* = 81, AUC: 0.696) (Figure 2b) exhibited more statistically robust performance (*p* = 0.001 for both), supported by larger cohort sizes. Posttreatment, all three biomarkers retained statistical significance with varying performance characteristics: CA125 exhibited a reduced AUC of 0.800 (*p* = 0.003), while maintaining a sensitivity of 80.00% and an improved specificity of 71.43%. CA19-9 remained stable with an AUC of 0.696 (*p* = 0.001), showing enhanced sensitivity (62.50%) but decreased specificity (70.73%). CEA displayed the most balanced posttreatment profile, with an AUC of 0.735 (*p* = 0.001), achieving 71.11% sensitivity and 73.91% specificity. Additionally, CRP demonstrated consistent significance at both pretreatment (*p* = 0.015) and posttreatment (*p* = 0.012) (Figure 2d), suggesting its potential utility for monitoring therapeutic response despite the limited sample size (*n* = 27). These findings establish CA125, CEA, and CA19-9 as cornerstone biomarkers for mortality risk prediction in patients with CRC receiving reduced-dose 5-FU, with CRP serving as a valuable complementary inflammatory indicator warranting further validation in larger cohorts.

**Figure 3:**
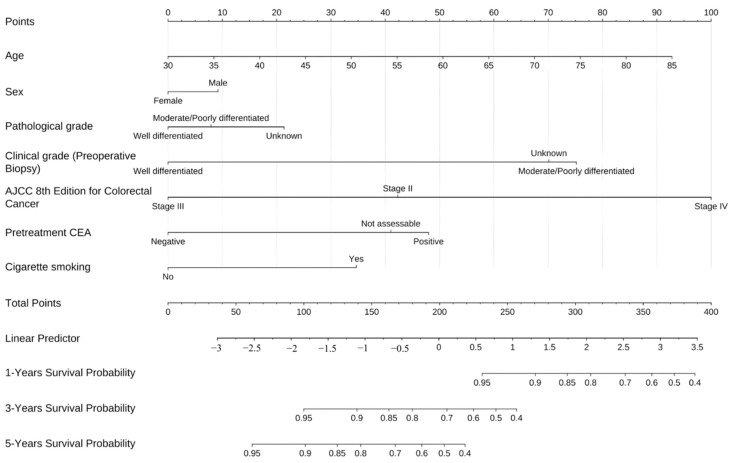
Nomogram for predicting overall survival in patients with colorectal cancer receiving reduced-dose 5-fluorouracil therapy.

Univariable Cox proportional hazards analysis (Table 5) identified several significant predictors of overall survival in patients receiving reduced-dose 5-FU. Clinical tumor differentiation was a strong prognostic factor, with moderately/poorly differentiated tumors associated with a higher risk of death than well-differentiated tumors (HR = 3.565, 95% CI: 1.478–8.597, *p* = 0.005). Disease stage had the strongest effect (stage IV vs. II: HR: 5.220, 95% CI: 1.734–15.708, *p* = 0.003). Elevated pretreatment CEA levels were also associated with worse survival (positive vs. negative: HR: 3.338, 95% CI: 1.721–6.476, *p* = 0.001). Factors associated with a higher mortality risk were unassessable perineural invasion (HR: 5.039, 95% CI: 1.712–14.830, *p* = 0.003) and cigarette smoking (HR: 1.936, 95% CI: 1.082–3.463, *p* = 0.026). Figure 3 presents a prognostic nomogram for estimating 1-, 3-, and 5-year overall survival in patients with CRC treated with reduced-dose 5-FU. The model was derived from a multivariable Cox proportional hazards framework and incorporates clinically and pathologically relevant predictors, including age, sex, clinical and pathological differentiation, AJCC 8th stage, pretreatment CEA levels, and cigarette smoking. In internal validation, the nomogram demonstrated acceptable discrimination (C-index = 0.812, bootstrap-corrected C-index = 0.835) with good agreement between predicted and observed survival on calibration plots at all three time horizons.

Table 6 summarizes patient characteristics stratified by risk categories derived from the prognostic nomogram, revealing three clinicopathological variables with significant associations across risk groups (all *p* < 0.001). Clinical tumor differentiation demonstrated a clear gradient distribution: well-differentiated tumors comprised 50% of the low-risk group compared with only 6.3% in the high-risk group, whereas moderately to poorly differentiated tumors predominated in the high-risk category (75%). The AJCC 8th edition staging system exhibited strong discriminatory power, with 100% of high-risk patients (*n* = 32) classified as stage IV, contrasting with no stage IV cases in the low-risk group. The medium-risk category showed an intermediate distribution, primarily comprising stage III cases (55%). Pretreatment CEA positivity increased stepwise with risk: 16% in low-risk, 61% in medium-risk, and 78% in high-risk groups, inversely mirrored by the corresponding negative rates of 84%, 32%, and 16%, respectively. This concordance among tumor differentiation grade, disease stage, and serum CEA elevation reinforces the nomogram’s capability to integrate multiple independent prognostic factors into an actionable risk stratification framework, with distant metastasis (stage IV) serving as the primary determinant of high-risk classification.

Table 7 summarizes the univariable and multivariable Cox proportional hazards regression analyses, identifying prognostic factors associated with overall survival in patients with CRC treated with reduced-dose 5-FU. In univariable analysis, cigarette smoking was significantly associated with increased mortality risk (HR = 1.936, 95% CI: 1.082–3.463, *p* = 0.026); however, this association lost statistical significance in the multivariable model, adjusting for confounders (HR: 1.891, 95% CI: 0.907–3.943, *p* = 0.089). Clinical tumor differentiation grade exhibited strong prognostic significance, with moderate to poorly differentiated tumors conferring substantially elevated mortality risk in both univariable (HR: 3.565, 95% CI: 1.478–8.597, *p* = 0.005) and multivariable analyses (HR: 3.985, 95% CI: 1.457–10.898, *p* = 0.007), establishing it as an independent adverse prognostic factor. Stage IV disease was strongly linked to unfavorable survival in the univariable analysis (HR: 5.220, 95% CI: 1.734–15.708, *p* = 0.003) but not in the multivariable model (HR: 2.894, 95% CI: 0.884–9.477, *p* = 0.079). Notably, pretreatment CEA positivity demonstrated consistent prognostic value in both models (univariable: HR: 3.338, 95% CI: 1.721–6.476, *p* < 0.001; multivariable: HR: 2.416, 95% CI: 1.161–5.026, *p* = 0.018). These findings confirm that the clinical tumor differentiation grade and elevated pretreatment CEA levels serve as independent prognostic biomarkers for overall survival in this patient population, independent of other clinicopathological variables.

Table 8 reports the results of proportional hazards assumption testing for the Cox regression model, revealing significant violations in three clinicopathological variables. Age exhibited time-dependent effects (χ^2^ = 7.061, df = 1, *p* = 0.008), indicating that its HRs varied significantly over the follow-up period. Cigarette smoking had the highest deviation from the proportional hazards assumption (χ^2^ = 12.155, df = 1, *p* < 0.001), suggesting substantial temporal variation in its influence on survival outcomes. Pathological tumor differentiation also violated the assumption (χ^2^ = 7.679, df = 2, *p* = 0.022), indicating nonconstant HRs across time intervals. The global test statistic (χ^2^ = 29.159, df = 10, *p* = 0.001) confirmed that the overall Cox model violated the proportional hazards assumption. These findings suggest that age, smoking status, and pathological differentiation exert time-varying prognostic effects rather than constant hazards throughout the observation period, warranting consideration of alternative analytical methods, such as stratified Cox regression or modeling of time-dependent covariates to ensure valid inference.

## 4. Discussion

This retrospective cohort study examined prognostic factors and biomarker performance in patients with CRC receiving reduced-dose 5-FU chemotherapy. In univariable analysis, three clinicopathological variables demonstrated significant associations with mortality: tumor differentiation grade, advanced disease stage, and elevated pretreatment CEA levels. Moderately differentiated tumors demonstrated a substantially elevated mortality risk (HR: 3.99). In multivariable Cox regression analysis, tumor differentiation grade (adjusted HR: 3.985, 95% CI: 1.457–10.898, *p* = 0.007) and pretreatment CEA levels (adjusted HR: 2.416, 95% CI: 1.161–5.026, *p* = 0.018) emerged as independent prognostic determinants. Advanced-stage disease exhibited a strong association with mortality in univariable analysis but not in the multivariable model (adjusted HR: 2.894, 95% CI: 0.884–9.477, *p* = 0.079), likely reflecting its correlation with poor differentiation and CEA elevation as well as the limited statistical power of this single-center cohort (*n* = 95) to disentangle highly correlated prognostic variables. Nonetheless, the clinical significance of disease stage in CRC prognosis remains undisputed, and our prognostic nomogram appropriately incorporates all three factors.

Among serum biomarkers, CA125 emerged as the most discriminative predictor of mortality (AUC = 0.929, *n* = 17), although this finding requires validation in larger cohorts due to the small sample size. CEA (AUC = 0.728, *n* = 91) and CA19-9 (AUC = 0.696, *n* = 81) demonstrated more robust performance with adequate sample sizes. Additionally, inflammation-related markers, particularly CRP, also exhibited significant predictive value. Collectively, these findings underscore the critical role of histopathological features and biomarker profiling in risk stratification for dose-adjusted chemotherapy populations.

Our results corroborate the established prognostic frameworks reported in large-scale international cohorts. The prominence of tumor differentiation grade aligns with findings from Luo et al. (2023) and Sudsawat Laohavinij et al. (2010), who identified histological grade as a pivotal survival determinant in CRC [15,16]. A 2024 systematic review also highlighted the prognostic significance of various biomarkers, including liquid biopsy markers, reinforcing these findings [17].

The mortality hazards associated with moderately differentiated tumors (univariable HR: 3.565; multivariable HR: 3.985) and advanced stages (univariable HR: 5.220) observed in our cohort are strongly consistent with recent large-scale analyses, which demonstrated histological grade and TNM stage as robust predictors across populations. Data from Taiwan’s national cancer registry similarly support these prognostic factors, confirming the external validity of our single-center study despite its smaller sample size [18,19].

We observed that CA125 was superior to CEA and CA19-9 in mortality risk discrimination. This result parallels Björkman et al. (2021) [13], who reported CA125 as a superior and independent prognostic factor compared with CEA in CRC. Similarly, Tutan et al. (2024) [20] confirmed that elevated CA125, CEA, and the C-reactive protein/albumin ratio were associated with significantly reduced survival and recurrence-free intervals, emphasizing the clinical utility of these markers in risk stratification [13,20]. However, given the small sample size with available CA125 measurements in our cohort, external validation in larger multicenter studies is essential before definitive clinical recommendations can be made.

Inflammation-related biomarkers, such as CRP, exhibited significant prognostic value, corroborating recent findings. Our results underscore the utility of incorporating dynamic immune markers into multimodal prognostic models, thereby enhancing the accuracy of survival predictions and informing more precise therapeutic decision-making [21,22,23].

The principal contribution of this study lies in demonstrating that robust prognostic associations remain detectable and clinically relevant, even in reduced-dose chemotherapy settings. Our data confirm that strategic dose modifications, based on careful clinical assessment (e.g., performance status and age), can preserve survival outcomes. While our study relied on empiric dose adjustment rather than real-time pharmacokinetic guidance, our findings align with the broader therapeutic goal of balancing efficacy and toxicity, consistent with principles seen in TDM-based studies [8,24]. The promising discriminative capacity of CA125 advances the rationale for incorporating multimarker panels, as advocated by Huang et al. (2016) and Zhang et al. (2025), to refine individualized risk assessment and therapeutic planning [25,26,27]. By providing institution-level evidence from a specialized treatment population, this work contributes pragmatic insights for clinical decision-making in resource-variable settings where dose adjustment is frequently necessary. From a practical perspective, these findings offer actionable guidance for everyday clinical decision-making, particularly when managing frail or elderly patients who require dose modifications. The strong predictive value of baseline CEA and CA19-9 suggests that these biomarkers should serve as critical indicators for establishing surveillance intensity; patients presenting with elevated baseline levels despite reduced-dose 5-FU initiation may warrant closer follow-up intervals and more rigorous monitoring for early signs of disease progression. Furthermore, the developed prognostic nomogram can be integrated into multidisciplinary team discussions to provide a visual and objective mortality risk estimation, thereby facilitating shared decision-making and helping to align treatment expectations between clinicians and patients. However, it is important to emphasize that this tool is currently preliminary. Its application should be considered complementary to clinical judgment, and rigorous external validation in larger, diverse cohorts is required to confirm its reliability before it is routinely adopted as a standard of care.

Several limitations warrant acknowledgment and suggest directions for future investigation. First, the modest sample size (*n* = 95) constrained statistical power for multivariable modeling. Additionally, the approach of modeling missing data as separate “Not assessable” categories contributed to sparse-data bias that compromised the reliability of fully adjusted logistic regression estimates, as evidenced by paradoxical associations contradicting univariable findings. Furthermore, the handling of missing data presents a methodological limitation. Key variables, including clinical grade, perineural invasion, and biomarkers, exhibited non-trivial missingness. To maximize the sample size, these were modeled as separate “Not assessable” categories rather than excluding cases or utilizing multiple imputation, given that the missingness mechanism was likely non-random (e.g., tests not ordered due to clinical urgency). However, this approach resulted in small cell counts within these subgroups, contributing to unstable estimates and wide confidence intervals, particularly in the logistic regression models. Consequently, interpretation of risk estimates for these “Not assessable” categories requires caution.

Consequently, we prioritized Cox proportional hazards regression for our primary prognostic analyses, which provided more stable and clinically interpretable estimates. Second, the retrospective single-center design introduces potential selection bias and limits generalizability to broader patient populations, particularly those receiving standard-dose regimens. Third, violations of the proportional hazards assumption in some covariates indicate that time-varying prognostic effects may not be adequately captured by constant HRs, suggesting the need for flexible parametric or time-dependent coefficient models in future analyses. Finally, the small analytic subset for CA125 (*n* = 17) necessitates cautious interpretation despite impressive AUC values and requires external validation in larger cohorts. Future multicenter prospective studies with adequate sample-to-variable ratios are warranted to validate optimal biomarker thresholds, investigate the temporal dynamics of prognostic factors using landmark analyses or restricted mean survival time methods, evaluate the incremental prognostic value of emerging liquid biopsy markers, and assess the clinical utility of integrated biomarker-clinical prediction models through decision curve analysis and prospective implementation studies.

## 5. Conclusions

Our findings revealed that tumor differentiation, disease stage, and pretreatment CEA levels are independent prognostic factors for overall survival in patients with CRC treated with reduced-dose 5-FU. Among serum biomarkers, CEA and CA19-9 had strong discriminatory power supporting their inclusion with clinicopathological parameters in comprehensive prognostic models. The validated prognostic nomogram demonstrates robust discrimination and calibration, supporting its use as a quantitative aid for personalized prognostication and shared treatment planning in this population.

## Figures and Tables

**Table 1:** Baseline characteristics of the study population.

Characteristics	Values
Total number of patients (N)	95
Sex (M/F)	55/40 (57.9%/42.1%)
Age (mean ± standard deviation (SD))	59.0 ± 10.7 (31–82)
Substance use	
Alcohol use	17(17.9%)
Betel nut chewing	8(8.4%)
Cigarette smoking	27(28.4%)
Primary Site	
C18 Colon	64(67.4%)
C19 Rectosigmoid Junction	9(9.5%)
C20 Rectum	22(23.1%)
Histology	
8140 Adenocarcinoma	95(100.0%)
Hepatitis	
HCV−/HBsAg−	66(69.5%)
HCV unknown /HBsAg−	5(5.3%)
HCV+/HBsAg−	7(7.4%)
HCV−/HBsAg+	17(17.9%)
Clinical grade (preoperative biopsy)	
Well-differentiated	24(25.3%)
Moderately differentiated	47(49.5%)
Poorly differentiated	3(3.2%)
Not assessable/Not available	21(22.1%)
Pathological grade	
Well-differentiated	7(7.4%)
Moderately differentiated	76(80.0%)
Poorly differentiated	5(5.3%)
Not assessable/Not available	7(7.4%)
Perineural invasion	
No	28(29.5%)
Yes	61(64.2%)
Not assessable/Not available	6(6.3%)
Lymphovascular invasion	
No	8(8.4%)
Yes	82(86.3%)
Not assessable/Not available	5(5.3%)
AJCC 8th Edition	
II	10(10.5%)
III	46(48.4%)
IV	39(41.1%)
ECOG	
0	29(30.5%)
1	60(63.2%)
2	4(4.2%)
3	2(2.1%)
Pretreatment CEA	
Positive	49(51.6%)
Negative	42(44.2%)
Not assessable/Not available	4(4.2%)
Obstruction	
No	81(85.3%)
Yes	14(14.7%)
Perforation	
No	94(98.9%)
Yes	1(1.1%)
Microsatellite Instability Detection	
No loss of nuclear expression	80(84.2%)
Loss of nuclear expression	4(4.2%)
No detection	11(11.6%)
BRAF	
Wild-type	26(27.4%)
Mutated	7(7.4%)
Not performed	62(65.2%)
KRAS	
Wild-type	60(63.2%)
Mutated	29(30.5%)
Not performed	6(6.3%)
NRAS	
Wild-type	72(75.8%)
Mutated	1(1.1%)
Not performed	22(23.2%)

**Table 2:** Univariable predictors of mortality in patients with colorectal cancer treated with reduced-dose 5-fluorouracil.

Characteristics	Univariable		
	OR	(95% CI)	*p* Value
Age	1.005	0.967–1.044	0.794
Sex (M/F)	0.843	0.373–1.896	0.680
Substance use			
Alcohol use (N/Y)	0.750	0.254–2.151	0.592
Betel nut chewing (N/Y)	0.425	0.073–1.870	0.263
Cigarette smoking (N/Y)	0.343	0.128–0.859	0.022 *
Clinical grade (Preoperative Biopsy)			
Well-differentiated	1		
Moderately differentiated/Poorly differentiated	4.452	1.605–13.653	0.004 *
Not assessable/Not available	3.117	0.940–11.133	0.063
Pathological grade			
Well-differentiated	1		
Moderately differentiated/Poorly differentiated	0.433	0.074–1.915	0.275
Not assessable/Not available	0.354	0.039–2.609	0.311
Hepatitis			
HCV−/HBsAg−	1		
HCV unknown /HBsAg−	1.486	0.271–9.426	0.644
HCV+/HBsAg−	1.186	0.416–3.427	0.748
HCV−/HBsAg+	1.365	0.308–6.530	0.679
Perineural invasion			
No	1		
Yes	0.615	0.246–1.498	0.286
Not assessable/Not available	3.434	0.633–34.941	0.161
Lymphovascular invasion			
No	1		
Yes	1.076	0.261–4.440	0.917
Not assessable/Not available	3.228	0.565–33.226	0.195
AJCC 8th Edition for Colorectal Cancer			
II	1		
III	0.582	0.150–2.400	0.440
IV	5.183	1.279–23.086	0.021 *
Pretreatment CEA			
Negative	1		
Positive	4.954	2.092–12.348	0.001 *
Not assessable/Not available	5.639	0.837–63.278	0.076
Obstruction (N/Y)	1.907	0.626–6.338	0.258
BRAF			
Wild-type	1		
Mutated	6.810	1.185–72.433	0.030 *
Not performed	1.623	0.653–4.167	0.299
KRAS			
Wild-type	1		
Mutated	1.735	0.713–4.345	0.226
Not performed	0.634	0.104–3.097	0.577

* Statistically significant (*p* < 0.05).

**Table 3:** Multivariable logistic regression analysis of predictors of mortality in patients with colorectal cancer receiving reduced-dose 5-FU.

Characteristics	Multivariable		
	OR	(95% CI)	*p* Value
Age	1.016	0.943–1.098	0.680
Sex (M/F)	0.950	0.165–5.157	0.952
Substance use			
Alcohol use (N/Y)	3.186	0.278–45.032	0.361
Betel nut chewing (N/Y)	105.092	2.436–7471.819	0.018 *
Cigarette smoking (N/Y)	0.005	0.000–0.108	0.003 *
Clinical grade			
Well-differentiated	1		
Moderately differentiated/ Poorly differentiated	36.153	3.299–949.513	0.010 *
Not assessable/Not available	43.371	1.962–2489.013	0.033 *
Pathological grade			
Well-differentiated	1		
Moderately differentiated/Poorly differentiated	0.053	0.001–1.078	0.079
Not assessable/Not available	0.001	0.000–0.128	0.012 *
Hepatitis			
HCV−/HBsAg−	1		
HCV unknown /HBsAg−	7.537	0.107–946.719	0.372
HCV+/HBsAg−	0.326	0.027–3.171	0.346
HCV−/HBsAg+	3.541	0.253–73.315	0.355
Perineural invasion			
No	1		
Yes	0.234	0.016–2.344	0.240
Not assessable/Not available	25.564	0.494–4781.408	0.148
Lymphovascular invasion			
No	1		
Yes	169.695	1.506–49,827.906	0.046 *
Not assessable/Not available	21.386	0.139–4056.178	0.217
AJCC 8th Edition for Colorectal Cancer			
II	1		
III	0.229	0.007–9.959	0.392
IV	6.262	0.364–269.056	0.252
Pretreatment CEA			
Negative	1		
Positive	0.109	0.013–0.633	0.023 *
Not assessable/Not available	0.361	0.002–71.085	0.704
Obstruction (N/Y)	4.844	0.235–148.629	0.327
BRAF			
Wild-type	1		
Mutated	91.049	1.686–19,396.700	0.055
Not performed	7.734	1.149–78.223	0.051
KRAS			
Wild-type	1		
Mutated	3.495	0.503–34.436	0.230
Not performed	0.030	0.001–0.600	0.030

Note: Because of sparse-data bias and quasi-complete separation, several estimates in this multivariable model show extreme odds ratios with wide confidence intervals and should be interpreted with caution. Univariable analyses and Cox regression models (Table 2 and subsequent analyses) provide more reliable prognostic indicators. * Statistically significant (*p* < 0.05).

**Table 4:** Comparison of biomarker sensitivity, specificity, and AUC in pre- vs. posttreatment setting.

Biomarker	N	Pretreatment					Posttreatment					*p* Value
		Sensitivity (%)	Specificity (%)	AUC (95% CI)	Youden’s Index Cut Point	*p* Value	Sensitivity (%)	Specificity (%)	AUC (95% CI)	Youden’s Index Cut Point	*p* Value	
AFP	68	65.62	19.44	0.552 (0.424–0.689)	1.845	0.445	46.88	27.78	0.616 (0.477–0.745)	2.335	0.090	0.158
CA125	17	90.00	85.71	0.929 (0.771–1.000)	13.155	0.001 *	80.00	71.43	0.800 (0.571–0.971)	12.540	0.003 *	0.235
CA19-9	81	55.00	85.37	0.696 (0.574–0.812)	29.715	0.001 *	62.50	70.73	0.696 (0.580–0.807)	11.550	0.001 *	0.987
CEA	91	73.33	69.57	0.728 (0.617–0.832)	5.565	0.001 *	71.11	73.91	0.735 (0.626–0.839)	3.945	0.001 *	0.850
eGFR	92	51.11	25.53	0.586 (0.469–0.706)	82.500	0.153	42.22	42.55	0.556 (0.438–0.677)	98.500	0.358	0.445
NLR	90	63.64	52.17	0.542 (0.419–0.664)	2.720	0.499	88.64	26.09	0.551 (0.433–0.663)	2.815	0.382	0.908
CRP	27	68.75	81.82	0.747 (0.528–0.926)	1.695	0.015 *	68.75	81.82	0.741 (0.534–0.909)	2.530	0.012 *	0.966
PSA	12	50.00	0.00	0.688 (0.250–1.000)	0.465	0.327	50.00	0.00	0.656 (0.250–1.000)	0.555	0.414	0.629

* Statistically significant (*p* < 0.05).

**Table 5:** Univariable Cox proportional hazard analysis of prognostic factors for overall survival in patients with colorectal cancer receiving reduced-dose 5-fluorouracil.

Characteristics	Hazard Ratio	95% CI	*p* Value
Age	1.014	0.986–1.042	0.346
Sex (M/F)	0.756	0.423–1.350	0.345
Substance use			
Alcohol use (Y/N)	1.387	0.686–2.803	0.362
Betel nut chewing (Y/N)	1.974	0.833–4.682	0.123
Cigarette smoking (Y/N)	1.936	1.082–3.463	0.026 *
Clinical grade			
Well-differentiated	1		
Moderately differentiated/ Poorly differentiated	3.565	1.478–8.597	0.005 *
Not assessable/Not available	2.313	0.854–6.261	0.099
Pathological grade			
Well-differentiated	1		
Moderately differentiated/ Poorly differentiated	0.798	0.313–2.030	0.635
Not assessable/Not available	0.743	0.177–3.122	0.685
Perineural invasion			
No	1		
Yes	1.863	0.922–3.764	0.083
Not assessable/Not available	5.039	1.712–14.830	0.003 *
Hepatitis			
HCV−/HBsAg−	1		
HCV unknown /HBsAg−	2.475	0.743–8.248	0.140
HCV+/HBsAg−	1.273	0.606–2.672	0.523
HCV−/HBsAg+	0.880	0.310–2.499	0.810
Lymphovascular invasion			
No	1		
Yes	1.146	0.407–3.228	0.797
Not assessable/Not available	3.517	0.858–14.423	0.081
CRC staging (AJCC 8th Edition)			
II	1		
III	0.760	0.244–2.360	0.634
IV	5.220	1.734–15.708	0.003 *
Pretreatment CEA			
Negative	1		
Positive	3.338	1.721–6.476	0.000 *
Not assessable/Not available	3.536	0.986–12.689	0.053
Obstruction (N/Y)	1.486	0.719–3.071	0.285
BRAF			
Wild-type	1		
Mutated	2.751	0.997–7.591	0.051
Not performed	1.489	0.726–3.057	0.278
KRAS			
Wild-type	1		
Mutated	1.636	0.903–2.963	0.104
Not performed	0.613	0.145–2.590	0.506

* Statistically significant (*p* < 0.05).

**Table 6:** Patient characteristics according to risk stratification derived from the prognostic nomogram.

Characteristics	Overall (N = 95) ^1^	Low Risk (N = 32) ^1^	Medium Risk (N = 31) ^1^	High Risk (N = 32) ^1^	*p*-Value ^2^
Age	59 (51–67)	58 (51–64)	59 (48–67)	61 (56–72)	0.309
Sex					0.749
Male	55 (58%)	17 (53%)	18 (58%)	20 (63%)	
Female	40 (42%)	15 (47%)	13 (42%)	12 (38%)	
Cigarette smoking	27 (28%)	6 (19%)	11 (35%)	10 (31%)	0.308
Pathological grade					0.835
Well-differentiated	7 (7.4%)	2 (6.3%)	2 (6.5%)	3 (9.4%)	
Moderate/Poorly differentiated	81 (85%)	29 (91%)	26 (84%)	26 (81%)	
Unknown	7 (7.4%)	1 (3.1%)	3 (9.7%)	3 (9.4%)	
Clinical grade					0.001 *
Well-differentiated	24 (25%)	16 (50%)	6 (19%)	2 (6.3%)	
Moderate/Poorly differentiated	50 (53%)	9 (28%)	17 (55%)	24 (75%)	
Unknown	21 (22%)	7 (22%)	8 (26%)	6 (19%)	
AJCC 8th Edition					0.001 *
Stage II	10 (11%)	3 (9.4%)	7 (23%)	0 (0%)	
Stage III	46 (48%)	29 (91%)	17 (55%)	0 (0%)	
Stage IV	39 (41%)	0 (0%)	7 (23%)	32 (100%)	
Pretreatment CEA					0.001 *
Negative	42 (44%)	27 (84%)	10 (32%)	5 (16%)	
Positive	49 (52%)	5 (16%)	19 (61%)	25 (78%)	
Not assessable	4 (4.2%)	0 (0%)	2 (6.5%)	2 (6.3%)	

^1^ Median (Q1, Q3); n (%); ^2^ Kruskal–Wallis rank sum test; Pearson’s Chi-square test; Fisher’s exact test; * Statistically significant (*p* < 0.05).

**Table 7:** Univariable and multivariate Cox proportional hazards regression analysis of prognostic factors for overall survival in patients with colorectal cancer receiving reduced-dose 5-fluorouracil therapy.

	Univariable Analysis			Multivariable Analysis		
Characteristic	HR	95% CI	*p* Value	HR	95% CI	*p* Value
Age	1.014	0.986–1.042	0.346	1.032	1.000–1.064	0.048
Sex						
Male	1					
Female	0.756	0.423–1.350	0.345	0.844	0.403–1.767	0.653
Cigarette smoking						
No	1					
Yes	1.936	1.082–3.463	0.026 *	1.891	0.907–3.943	0.089
Pathological grade						
Well-differentiated	1					
Moderate/Poorly differentiated	0.798	0.313–2.030	0.635	1.157	0.388–3.450	0.794
Unknown	0.743	0.177–3.122	0.685	1.479	0.300–7.298	0.631
Clinical grade						
Well-differentiated	1					
Moderate/Poorly differentiated	3.565	1.478–8.597	0.005 *	3.985	1.457–10.898	0.007 *
Unknown	2.313	0.854–6.261	0.099	3.630	1.207–10.922	0.022
AJCC 8th Edition						
Stage II	1					
Stage III	0.760	0.244–2.360	0.634	0.460	0.138–1.533	0.206
Stage IV	5.220	1.734–15.708	0.003 *	2.894	0.884–9.477	0.079
Pretreatment CEA						
Negative	1					
Positive	3.338	1.721–6.476	0.001 *	2.416	1.161–5.026	0.018 *
Not assessable	3.536	0.986–12.689	0.053	2.127	0.496–9.114	0.310

CI: Confidence interval; HR: Hazard ratio.* Statistically significant (*p* < 0.05).

**Table 8:** Proportional hazards assumption testing for the Cox model.

Variable	Chi_Square	df	*p*_Value
Age	7.061	1	0.008 *
Sex	1.346	1	0.246
Cigarette smoking	12.155	1	0.001 *
Pathological grade	7.679	2	0.022 *
Clinical grade	1.194	2	0.551
AJCC 8th Edition for Colorectal Cancer	1.244	2	0.537
Pretreatment CEA	0.287	1	0.592
GLOBAL	29.159	10	0.001 *

* Statistically significant (*p* < 0.05).

## Data Availability

The datasets generated and/or analyzed during the current study are not publicly available, due to the privacy of the enrolled patients, but these may be requested from the corresponding author, upon reasonable request.

## Abbreviations

The following abbreviations are used in this manuscript:

BRAF	B-Raf proto-oncogene, serine/threonine kinase
KRAS	Kirsten rat sarcoma viral oncogene homolog
NRAS	Neuroblastoma RAS viral oncogene homolog
CRP	C-reactive protein
NLR	Neutrophil-to-Lymphocyte Ratio
CEA	Carcinoembryonic antigen
CA19-9	Carbohydrate antigen 19-9
CA125	Cancer antigen 125
AFP	Alpha-fetoprotein
